# GRP75 modulates oncogenic Dbl-driven endocytosis derailed via the CHIP-mediated ubiquitin degradation pathway

**DOI:** 10.1038/s41419-018-1039-2

**Published:** 2018-09-24

**Authors:** Xiuran Niu, Linjia Su, Shanshan Qi, Zhihui Gao, Qing Zhang, Sihe Zhang

**Affiliations:** 1 Department of Cell Biology, School of Medicine, Nankai University, Tianjin, China; 2 Department of Clinical Laboratory, Cancer Hospital of Tianjin Medical University, Tianjin, China

## Abstract

Chaperone-assisted proteasome degradation of oncogenic protein acts as an upstream signal controlling tumorigenesis and progression. The understanding of the co-regulation of chaperone and oncoprotein of endocytosis pathways is extremely limited. In this study, we showed for the first time that proto-Dbl (*dbl* proto-oncogene product) is co-enriched with mitochondrial chaperone GRP75 in endocytosis vesicles from ovarian cancer cells. onco-Dbl, produced by oncogenic mutation/degradation of proto-Dbl, markedly enhanced cellular macropinocytosis but suppressed clathrin-mediated endocytosis and clathrin-independent endocytosis pathways, presenting a derailed endocytosis phenotype. GRP75 was associated with proto-Dbl inside cells and modulated Dbl-driven endocytosis derailed by a co-regulatory mode. In spite of not being a component of the Hsc70/Hsp90/proto-Dbl complex, the degradation of proto-Dbl was promoted by GRP75 through the CHIP-mediated ubiquitin–proteasome pathway, of which GRP75 acts as a cooperator with CHIP but also acts as a competitor to Hsc70 and Hsp90 in the multiple chaperones-assisted pro-folding/pro-degradation machinery. Knockdown or inhibition of GRP75 attenuated proto-Dbl degradation and reduced the onco-Dbl level, which differentially impaired Rho GTPases activation and therefore shifted the endocytosis-derailed phenotype. Our data uncovered a novel GRP75-Dbl endocytosis regulatory axis and provided an alternative using chaperone inhibitor to shut down the oncoprotein-driven endocytosis derailment mechanism.

## Introduction

Abnormal membrane and vesicle trafficking constitute a derailed endocytosis phenotype, which has emerged as a multifaceted hallmark of cancer cells^1–3^. The derailed endocytosis highly stimulates cancer cell uptake of certain nutrients to sustain their growth and proliferation in hostile microenvironments, and this characteristic also develops an endocytosis-mediated defense system against therapeutic macromolecules^1,3–5^. Thus, a clear understanding of the endocytosis-derailed mechanism is a major challenge in tumor cell biology with implications for the development of endocytosis pathway-selective drug delivery^4^.

Increasing evidence shows that derailed endocytosis is driven by various oncogenic alterations^2^, including oncogene amplification resulting in overexpression of oncoproteins. Accumulation of oncoproteins activates downstream Rho GTPases, such as the three best-characterized Cdc42, Rac1, and RhoA, which induce distinct endocytosis changes^6^. In most cases, the activation of Rho GTPases is facilitated by a family of oncoproteins known as Dbl (first discovered in human diffuse B-cell lymphoma) guanine nucleotide exchange factors (GEFs)^7–9^. Oncogenic activation of proto-Dbl, the dbl proto-oncogene product, occurs through loss of the amino-terminal residues, producing a constitutively active onco-Dbl with high oncogenic potential. As both onco- and proto-Dbl contain the Dbl homology (DH) and pleckstrin homology (PH) domains required for GEF activity, it is thought that the amino terminus of proto-Dbl maintains the protein in an auto-inhibitory status via the chaperone-mediated intramolecular regulation mode^10,11^.

The chaperone/co-chaperone-based triage balance between protein folding and degradation controls the steady state level of oncogenic proteins^12,13^. Molecular chaperones Hsp70 and Hsp90, co-chaperones HOP (Hsp70/Hsp90-organizing protein), and CHIP (carboxyl terminus of Hsc70/Hsp70/90-interacting protein) are the central players determining this balance^14^. HOP binds to Hsp70 and Hsp90, thus forming a pro-folding chaperone complex, which facilitates entry of the substrate from the Hsp70 complex into the Hsp90 complex. In contrast, the recruitment of CHIP to the chaperones forms a pro-degradation complex, which leads to substrate degradation through the ubiquitin–proteasome system^15^. The folding and degradation machinery cannot actually coexist in one complex. The fate of an oncogenic protein is dictated by the chaperone/co-chaperone combinations and the cooperating or competing relations they establish^12,13,16,17^. Although previous reports have documented the regulatory role of the Hsc70/Hsp90/CHIP complex in ubiquitin-mediated degradation of proto-Dbl^10,18^, the exact details dictating the stabilization versus the degradation process are incompletely understood. Indeed, binding with Hsp90 dictates the stabilization of proto-Dbl, while CHIP recruitment directs the protein to ubiquitination degradation. However, the molecular basis of these regulatory interactions is largely unknown, and it is unclear whether other (co) chaperones are involved in these interactions and thus modulate the degradation rate of proto-Dbl.

Glucose-regulated proteins (GRPs) are stress inducible chaperones mainly residing in the endoplasmic reticulum (ER) and the mitochondria. Recent advances revealed that the GRPs serve distinct functions from the related heat shock proteins in cancer cells, and they can be actively translocated to other cellular locations and assume novel functions including endocytosis signal control^19^. For instance, the ER-resident GRP78 (BiP/HspA5) was reported to translocate on the cell surface and function as a co-receptor in a lipid raft or caveolae-mediated endocytosis of several viruses and matrix proteins^14,15,19^. The mitochondria-resident GRP75 (mortalin/HspA9) was shown to bind with certain cytokines (FGF-1) or cytokine receptors (IL-1R1, mannose receptor) in cytosol^20–22^, or bind with the complement the C5b-9 complex on the cell surface^23^. We previously accidentally found that GRP75 functions as a key constituent in heparan sulfate proteoglycan (HSPG)-mediated and membrane raft-associated endocytosis vesicles^24^. More recently, we further found that GRP75 promotes clathrin-independent endocytosis (CIE) but inhibits clathrin-mediated endocytosis (CME) through the RhoGTPases concurrent activation mechanism^25^, and this derailed cellular endocytosis phenotype was controlled by the cell-cycle-dependent expression variation of GRP75 in cancer cells^26^. These evidences collectively indicate that GRP75 plays an active role in endocytosis processes depending on its specific subcellular localizations, and implies that its trans-localization to outside the mitochondria results in its collaboration with different binding partners while exerting an endocytosis regulatory function. In this study, we found that proto-Dbl is co-enriched with GRP75 in endocytic vesicles derived from onco-Dbl high-expressing ovarian cancer cells. We further showed that GRP75 functions as a novel regulator of the onco-Dbl-driven endocytosis-derailed phenotype. Importantly, we found that GRP75 promotes proto-Dbl degradation via the CHIP-mediated ubiquitin–proteasome pathway (UPP), and GRP75 expression modulates onco-Dbl-triggered Rho GTPases activation, which causes the striking derailment of endocytosis pathways inside ovarian cancer cells.

## Results

### Co-enriched proto-Dbl with GRP75 in endocytosis vesicles

**Fig. 1:**
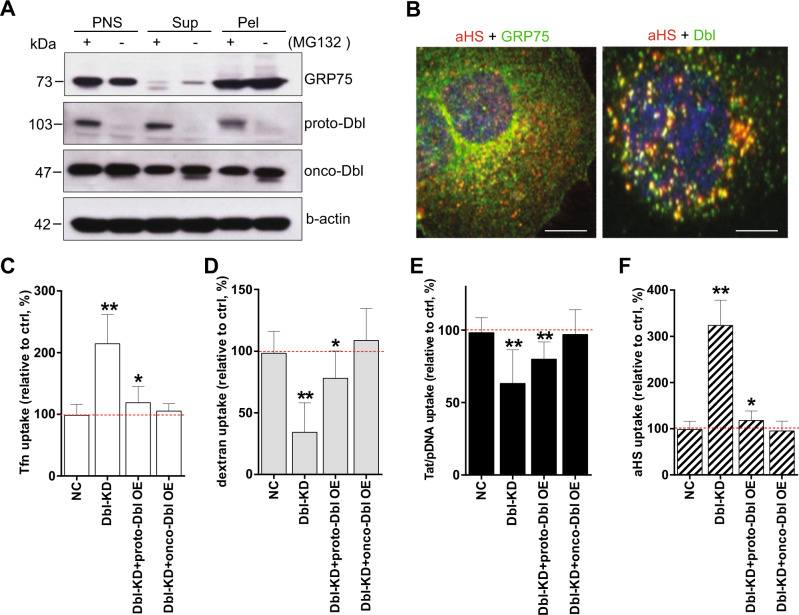
Dbl co-enriched with GRP75 in vesicles and functions as an endocytosis derailer. **a** SKOV-3 cells were incubated with scFv-αHSM nanoparticles at 37 °C for 1 h uptake. Endocytic vesicles were isolated and purified by the magnetic pick-pen method as described in “Materials and methods” (Supplementary Fig. 1). The PNS (post-nuclear supernatant. cell nuclei, and debris were removed), Sup (the supernatant was washed out during magnetic purification) and Pel (magnetic separation of vesicular pellets) fractions from cells pre-treated with/without MG132 (50 μM, 6 h) were subjected to SDS-PAGE, and vesicular proteins were analyzed by Western blot using the indicated Abs. **b** MG132-pre-treated SKOV-3 cells were incubated for 1 h at 37 °C with scFv-αHS-AF647 complexes, and the endogenous GRP75 and Dbl were stained by primary Abs followed by the AF488-conjugated secondary Ab. The co-localization of scFv-αHS complexes with indicated proteins was visualized using confocal microscopy. **c**, **d**, **e**, **f** Dbl knockdown SKOV-3 stable cell lines (Dbl-KD; D1, D2, D3) were produced by the CRISPR–CAS9 system. The uptakes of Tfn-AF647, Dextran-Rhodamine, scFv-αHS-AF647, and Tat/pGL3-YOYO-1 complexes in untransfected SKOV-3 cells (Ctrl, set as 100%), in Dbl-KD (D3) cell populations, or in proto- or onco-Dbl overexpression-rescued cell populations were immediately quantified by FACS. 10,000 cells were counted per sample with triplicate samples per transfection for each experiment, *n* = 3. Statistically significant differences compared with the uptakes in Scramble-sgRNA stable cell lines (NC) are shown: **P < 0.01, *P < 0.05

We previously found that mitochondrial chaperone GRP75 is present on cancer cell surfaces and enriched in HSPG-induced endocytic vesicles with an uptake regulatory function^24–26^. However, it remains ill-defined whether other vesicular proteins are also co-enriched with GRP75 and act as functional components. Again, we took advantage of single-chain variable fragment anti-HS-conjugated superparamagnetic nanoparticles (scFv-αHS_M_) as internalizing ligands^24^. Magnetic separation of mechanically disrupted SKOV-3 cells (Supplementary Fig. 1) that yielded a population of intact scFv-αHS_M_-containing vesicles to identify the proteins co-enriched with GRP75. 1D gel electrophoresis followed by LC-MS/MS analysis showed the enrichment of several proteins in vesicular preparations (Supplementary Fig. 2). Interestingly, one of these proteins was proto-Dbl. Western blot compared the protein composition of isolated vesicles and showed that proto-Dbl was significantly co-enriched with GRP75 in endocytosis vesicles derived from MG132 pre-treated SKOV-3 cells (Fig. 1a). In contrast, the co-enrichment of proto-Dbl with GRP75 was not observed in cellular vesicles without MG132 treatment. Confocal imaging analysis further showed that GRP75 and Dbl were localized in scFv-αHS_F_ (fluorophore-labeled scFv-αHS complexes)-containing vesicular compartments (Fig. 1b). However, significant co-localization of GRP75 with proto-Dbl was only observed in MG132 pre-treated cells (Fig. 3e).

Dbl has an oncogenic GEF function immediately upstream of Rho GTPase signaling and regulates various physiological processes^7,8,27,28^. Recently, more studies highlighted the oncogene-driven endocytosis-derailed phenotype in cancer cells^1,2,29^. To determine whether oncogenic Dbl can induce cellular endocytosis derailment, various endocytosis pathway markers were used to check their uptake changes. Flow cytometry results showed that Dbl knockdown significantly decreased the uptake of the macropinocytosis marker dextran (average decrement 65%) but increased the CME marker Tfn uptake (average increment 110%) in SKOV-3 cells (Fig. 1c, d). These sharply contrasted uptake changes were only partially rescued by proto-Dbl overexpression but completely rescued by onco-Dbl overexpression (Fig. 1c, d). Accordingly, the Dbl knockdown/overexpression-induced uptake changes of the Tat/pGL3 nano-complex (Fig. 1e), which are frequently internalized via the macropinocytosis pathway^30,31^, were similar to those of dextran. Unexpectedly, we detected little uptake of the lipid-raft marker CtxB in SKOV-3 cells. In addition, the uptake of scFv-αHS_F_ complexes, which follow the CIE pathway in cancer cells^24–26,32^, was changed similarly to Tfn uptake after the modulations on Dbl expression (Fig. 1f). Together, these data indicate that proto-Dbl is co-enriched with GRP75 in endocytic vesicles and oncogenic Dbl functions as a novel endocytosis derailer.

### Onco-Dbl-driven endocytosis derailment is modulated by GRP75

**Fig. 2:**
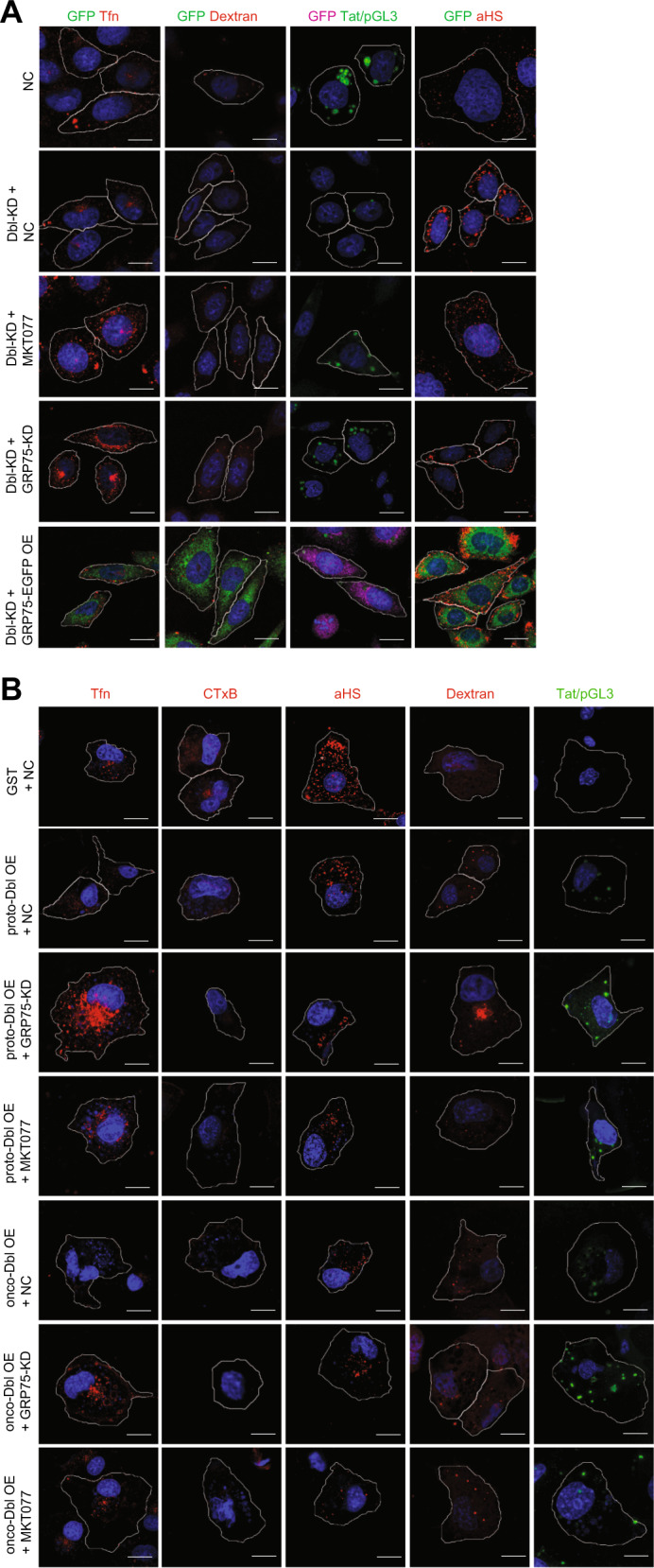
Oncogenic Dbl-driven endocytosis derailment is modulated by GRP75. **a** Dbl-KD (D3) and NC SKOV-3 stable cell lines were transfected either with the GRP75-targeting shRNA Lentivirus (GRP75-KD), or with GRP75-pEGFP plasmids, or further treated with its chemical inhibitor MKT077 (40 µM, 12 h). After culture for 36 h, cells had Tfn-AF647, CTxB-AF647, Dextran-Rhodamine, scFv-αHS-AF647, and Tat/pGL3-YOYO-1 complexes added for 37 °C uptake as described in “Materials and methods”. The uptakes of each drug were determined by confocal imaging analysis, and representative images from three independent experiments are shown. Scale bar: 20 µm. The uptake variability of indicated transfections/treatments in single cell populations is shown in Supplementary Fig. 3 A–D; **b** GRP75 knockdown Cos-7 stable cell lines (GRP75-KD; G1, G2, G3) were produced by the CRISPR–CAS9 system. GRP75-KD (G3), NC Cos-7 stable cell lines, and MKT077-treated Cos-7 cells were transfected with either GST-proto-Dbl or GST-onco-Dbl plasmids. The incubation of fluorescent-labeled drugs and confocal-based uptake analysis were as described as above. The uptake variability in single cell populations is shown in Supplementary Fig. 3e–i

Seeing that both Dbl (Fig. 1) and GRP75^24–26^ exert regulatory functions on endocytosis, their co-enrichment in endocytic vesicles prompted us to investigate whether the derailed cellular endocytosis phenotype was modulated by these two proteins in a dependent manner. Confocal imaging analysis showed that GRP75 knockdown or inhibition synergistically increased the uptakes of the CME marker Tfn (average increments 140%, 50%) in Dbl knockdown SKOV-3 cells (Fig. 2a, Supplementary Fig. 3a). In contrast, the knockdown or inhibition of GRP75 antagonistically decreased scFv-αHS_F_ uptake (average decrements 125%, 120%) but increased the uptakes of the macropinocytosis marker dextran (average increments 60, 43%) and the Tat/pGL3 nano-complex (38, 25%) in Dbl knockdown SKOV-3 cells (Fig. 2a, Supplementary Fig. 3b, c, d). Conversely, ectopically overexpression of GRP75 in Dbl knockdown SKOV-3 cells significantly reversed the uptake changes of these drugs (Fig. 2a, Supplementary Fig. 3a, b, c, d). In addition, these dependent actions of Dbl and GRP75 on endocytosis regulation were also observed in flow cytometry results (Supplementary Fig. 4a–d). These results suggest that the expression level of GRP75 affects Dbl-induced endocytosis derailment in SKOV-3 cells.

To further verify this point, GRP75 knockdown or inhibited Cos-7 cells were transfected with proto- or onco-Dbl plasmids for overexpression, and the uptake changes of indicated drugs were investigated. Confocal imaging results showed that proto-Dbl or onco-Dbl overexpression significantly decreased the uptakes of Tfn (average decrements 20, 65%), CtxB (average decrements 35, 60%) and scFv-αHS_F_ (average decrements 55, 70%), but increased the uptakes of dextran (average increments 40, 70%) and the Tat/pGL3 nano-complex (average increments 5, 45%) in NC Cos-7 cells (Fig. 2b, Supplementary Fig. 3e–i). The knockdown or inhibition of GRP75 antagonistically increased the uptakes of Tfn (70 or 25%, and 50 or 40% in average increments), synergistically increased the uptakes of dextran (30 and 50% or 25% in average increments) and Tat/pGL3 nano-complex (80 or 40%, and 15 or 20% in average increments), but synergistic inhibited the uptakes of CtxB (45 or 35%, and 25 or 15% in average decrements) and scFv-αHS_F_ (25 or 23%, and 20 or 20% in average decrements) in proto- or onco-Dbl overexpressed Cos-7 cells (Fig. 2b, Supplementary Fig. 3e–i). Flow cytometry results again confirmed this dependent regulatory effect between GRP75 and Dbl acting on distinct endocytosis pathways (Supplementary Fig. 4e–i). Obviously, the oncogenic Dbl-driven derailed endocytosis is essentially modulated by the GRP75 expression level.

### GRP75 is not a component in the Hsc70/Hsp90/proto-Dbl complex

**Fig. 3:**
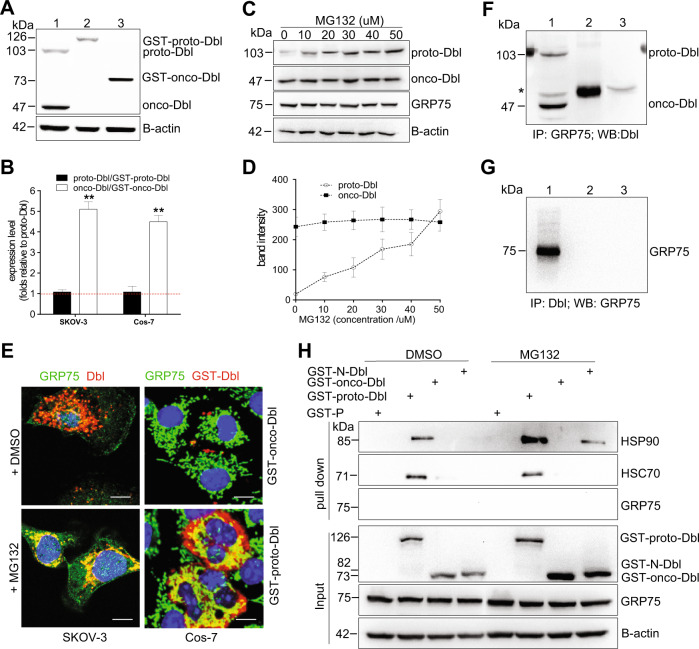
GRP75 is not a component in the Hsc70/Hsp90/proto-Dbl complex. **a** Western blot determined the steady-state level of endogenous Dbl in SKOV-3 cells (lane 1) and exogenously expressed GST-proto-Dbl (lane 2) or GST-onco-Dbl (lane 3) in transfected Cos-7 cells; **c** SKOV-3 cells were treated with MG132 (6 h) at indicated concentrations for inhibition of the ubiquitin–proteasome pathway. The degradation of proto-Dbl was analyzed by Western blot; Representative blot results from three independent experiments are shown in **a**, **c**. Protein brands were quantified by Image J software, and the expression ratio or variation of Dbl proteins were quantified and are shown in **b**, **e** SKOV-3 cells and transfected Cos-7 cells were stained by anti-GRP75 Ab (anti-rabbit AF488 as 2nd Ab) together with anti-Dbl Ab (or anti-GST Ab and anti-mouse AF647 as 2nd Ab). The subcellular co-localization of GR75 with endogenous Dbl or with exogenous GST-proto-Dbl/onco-Dbl was analyzed by confocal imaging. Endogenous Dbl and GRP75 were respectively co-immunoprecipitated (co-IP) by mouse anti-GRP75 Ab (lane 2) (**f**) and rabbit anti-Dbl Ab (lane 2) (**g**) in SKOV-3 cells. Proteins precipitates together with cell lysates (lane 1) were subjected to Western blot analysis using the indicated Abs. The isotype-matched IgG (lane 3) was used as the control. The asterisk points to an unspecific band when blotting with anti-Dbl Abs (proto-Dbl, 103 kDa, and onco-Dbl, 47 kDa); **h** Cos-7 cells were transfected with different Dbl constructs (as described in “Materials and methods”) followed by MG132 (25 μM, 6 h) treatment (DMSO as control). Cells were lysed 48 h post-transfection, and the lysates were subjected to GST pull-down. The precipitates were resolved by SDS-PAGE, and the co-pull-down protein was blotted using the indicated Abs. GST-P: pEBG-GST plasmid. GST-N-Dbl: N-terminal cloned Dbl (residues 1–498) into the pEBG-GST plasmid

Previous studies showed proto-Dbl is a client protein kept at an auto-inhibitory status by binding with chaperones Hsc70 and Hsp90^10,18^. To understand the molecular basis of Dbl-driven derailed endocytosis modulated by GRP75, we first checked the steady-state level of endogenous Dbl in SKOV-3 cells and exogenous GST-Dbl in transfected Cos-7 cells. Western blot results showed that oncogenic forms of Dbl exist at a markedly higher level than its proto-forms (Fig. 3a, b). When SKOV-3 cells and transfected Cos-7 cells were treated with proteasome inhibitor MG132, the protein levels of endogenous proto-Dbl and exogenous GST-proto-Dbl were both increased (Fig. 3c, d, h). These results verified previous claims that proto-Dbl is a short-lived protein due to the rapid degradation by the proteasome pathway^10,18^. To explore subcellular localization relationship between GRP75 and Dbl, cells treated with/without MG132 were stained by indicated Abs and subjected to confocal imaging. Markedly co-localization of GRP75 with exogenous GST-proto-Dbl (or with endogenous proto-Dbl) was observed in transfected Cos-7 cells (or in MG132-pre-treated SKOV-3 cells), whereas no/weak co-localization of GRP75 with onco-Dbl/GST-onco-Dbl was observed (Fig. 3e). This result implies that GRP75 may prefer to associate with proto-Dbl rather than with onco-Dbl.

To clarify whether GRP75 is involved in maintaining the Hsc70/Hsp90/proto-Dbl complex by the protein-binding mode, endogenous Dbl and GRP75 were co-immunoprecipitated (co-IP) in a bi-directional way. Co-IP results showed that neither proto-Dbl nor onco-Dbl directly bind to GRP75 in SKOV-3 cells (Fig. 3f, g). To further examine if GRP75 is associated with the Hsc70/Hsp90/proto-Dbl complex, exogenous-expressed GST-Dbl in transfected Cos-7 cells was pulled-down and the co-pull-down proteins were analyzed. As was expected, Hsc70 and Hsp90 proteins, but no GRP75, were detected in the co-pull-down precipitates (Fig. 3h). The non-involvement of GRP75 in the Hsc70/Hsp90/proto-Dbl complex was evidenced again even the proteasome-mediated degradation of proto-Dbl was totally inhibited (Fig. 3h). These results indicate that GRP75 can associate with proto-Dbl but is not a component in the Hsc70/Hsp90/proto-Dbl complex.

### GRP75 promotes proto-Dbl degradation by the CHIP-mediated UPP

**Fig. 4:**
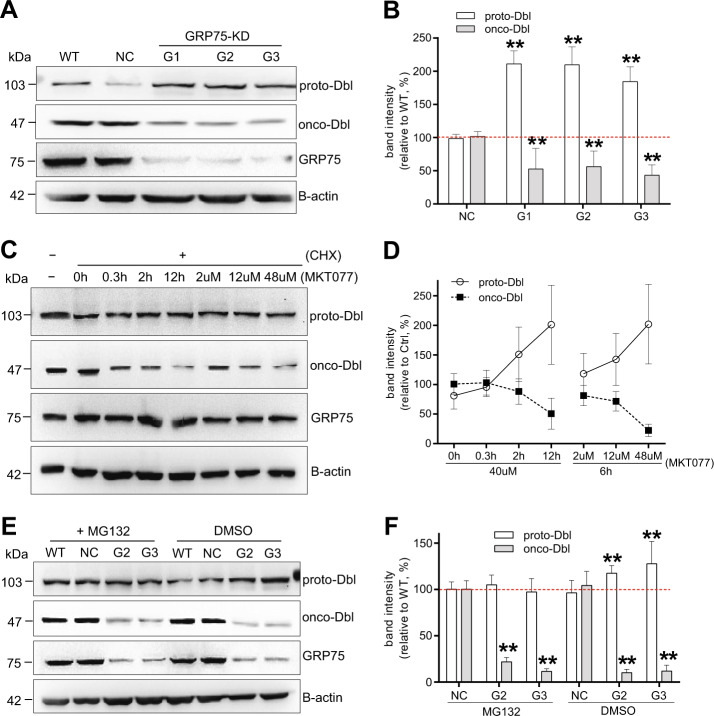
GRP75 inhibition attenuates the degradation of proto-Dbl. **a** GRP75 knockdown stable cell lines (GRP75-KD: G1, G2, G3) were produced from SKOV-3 cells (WT) by the CRISPR–CAS9 system. pLenti-CRISPR v2 plasmid transfected SKOV-3 cells was set as the control (NC). The protein levels of endogenous proto-Dbl, onco-Dbl and GRP75 were determined by Western blot using the indicated Abs. **c** SKOV-3 cells were treated with/without CHX (100 µg/ml) for 12 h to inhibit de novo protein synthesis, and the GRP75 inhibitor MKT077 was added either in a time-dependent or in a concentration-dependent manner. The protein levels of endogenous proto- and onco-Dbl were analyzed by Western blot. **e** GRP75-KD stable cell lines (G2, G3) were pre-treated with the UPP inhibitor MG132 (50 μM, 6 h) before harvest. DMSO treatment was used as the control. The protein level changes of proto- and onco-Dbl after MG132 treatment was analyzed by Western blot. Representative blot results from three independent experiments are shown in **a**, **c**, **e**, and corresponding quantitation data (determined similarly as above) are shown in **b**, **d**, **f**. Statistically significant differences compared with corresponding NC groups are shown: **P < 0.01

The proteasome pathway dependent association of proto-Dbl with GRP75 (Fig. 1a, 3e) but not directly involving GRP75 in its auto-inhibitory complex (Fig. 3f–h) raises the possibility that proto-Dbl is subjected to degradation by GRP75 in an indirect regulatory mode. To examine this possibility, we checked the changes of proto-Dbl degradation in GRP75 knockdown/inhibited SKOV-3 cells. GRP75 knockdown did slow down the degradation of proto-Dbl but markedly increased its protein level (Fig. 4a, b). When these GRP75 knockdown cells were treated with MG132, the increment of proto-Dbl was diminished (Fig. 4e, f). The attenuated degradation of proto-Dbl resulted in the onco-Dbl level correspondingly decreasing. To exclude the protein turnover effect on Dbl level changes, SKOV-3 cells were treated with the protein synthesis inhibitor cycloheximide together with the GRP75 chemical inhibitor MKT077, and the degradation changes of proto-Dbl were rechecked. Again, GRP75 inhibition not only attenuated the degradation of proto-Dbl but also significantly increased its protein level (Fig. 4c, d). These results suggest that GRP75 promotes proto-Dbl degradation via the proteasome pathway.

**Fig. 5:**
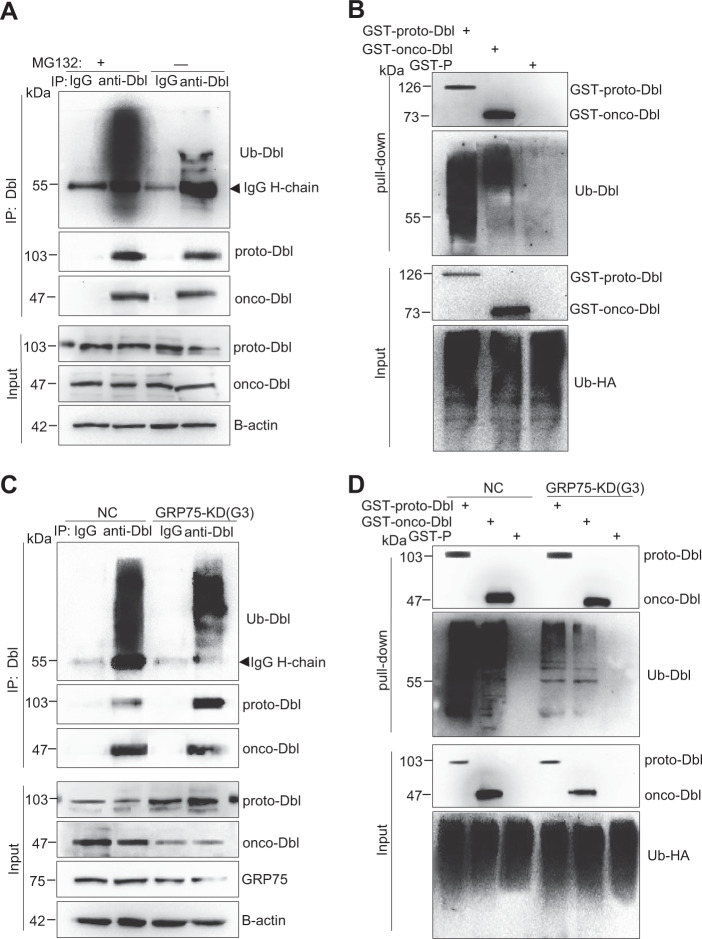
GRP75 knockdown weakens proto-Dbl ubiquitin modification. **a** Endogenous Dbl was immunoprecipitated by rabbit anti-Dbl Ab from the lysate of SKOV-3 cells pre-treated with/without MG132 (50 μM, 6 h). Isotype-matched rabbit IgG was used as the control. Dbl and ubiquitinated proto-Dbl from the precipitates were determined by Western blot using rabbit anti-Dbl Ab and mouse anti-ubiquitin (Ub) Ab, respectively. **b** Cos-7 cells were first co-transfected with GST-Dbl constructs and HA-Ub plasmids for 40 h culture, then treated with MG132 (25 μM) for 6 h. Exogenous Dbl was pull-down from cell lysate by glutathione sepharose 4B, ectopically expressed and ubiquitinated GST-Dbl was determined by Western blot using mouse anti-GST Ab and mouse anti-HA Ab, respectively. **c** GRP75-KD (G3) and NC SKOV-3 stable cell lines were treated with MG132 before harvest. After immunoprecipitation by rabbit anti-Dbl Ab, the Dbl protein level, and its ubiquitin modification were evaluated by Western blot using rabbit anti-Dbl Ab and mouse anti-Ub Ab, respectively. The isotype-matched IgG was used as the control; **d** GRP75-KD (G3) and NC Cos-7 stable cell lines were co-transfected with GST-Dbl constructs and HA-Ub plasmids followed by MG132 treatment as described above. The ectopic expressed GST-Dbl proteins and their ubiquitination changes were analyzed by Western blot using rabbit anti-GST Ab and mouse anti-Ub Ab, respectively. Representative blot results from three independent experiments are shown

Previous studies showed that proto-Dbl is frequently ubiquitinated and that this modification triggers its rapid degradation^18^. To examine whether proto-Dbl is targeted for degradation by GRP75-mediated ubiquitin modification, the ubiquitination level of Dbl proteins was checked in GRP75 knockdown cells. Immunoprecipitated proto-Dbl from MG132-pre-treated SKOV-3 cell lysates displayed an intense ubiquitinated band (Fig. 5a). Notably, this intense ubiquitinated band was significantly weakened when GRP75 expression was essentially inhibited or knocked-down (Fig. 5c). To confirm this observation, exogenously expressed GST-proto-Dbl and -onco-Dbl from myc-Ub-co-transfected Cos-7 cells were immunoprecipitated, and a similar ubiquitination-diminished pattern was observed when GRP75 was stably knocked-down (Fig. 5b, d). These results indicate that proto-Dbl is targeted for degradation by GRP75-mediated ubiquitination.

**Fig. 6:**
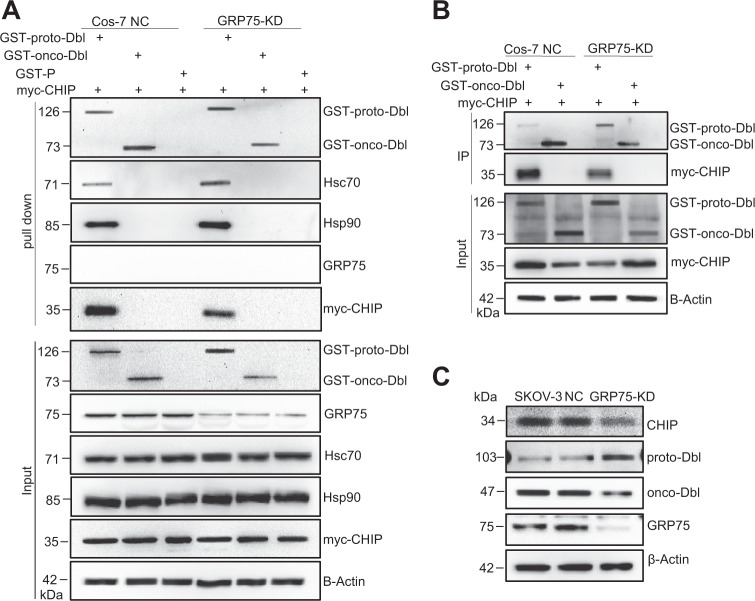
GRP75 knockdown impairs CHIP expression and its binding to proto-Dbl. **a** GRP75 KD (G3) and NC Cos-7 stable cell lines were co-transfected with GST-Dbl constructs and myc-CHIP plasmids. Exogenous GST-proto-Dbl were pull-down from cell lysate by mouse anti-GST Ab, and co-pull-down CHIP, Hsc70, and HSP90 were evaluated by their corresponding Ab, respectively. **b** GRP75 KD (G3) Cos-7 stable cells were co-transfected with GST-Dbl constructs and myc-CHIP plasmids. 48 h post-transfection, exogenous GST-Dbl were immunoprecipitated from cell lysates by mouse anti-GST Ab, and co-immunoprecipitated CHIP was evaluated by rabbit anti-Myc Ab. The lysate of the NC Cos-7 stable cell line was included as the control (NC). **c** The expression level of endogenous CHIP, proto-, and onco-Dbl among SKOV-3, GRP75 KD (G2), and NC SKOV-3 stable cell lines were compared by Western blot. Representative blot results from three independent experiments are shown

The chaperone-controlled ubiquitin–proteasome system is strictly dictated by the triage decision, a delicate interplay between multiple chaperones, co-chaperones, and ubiquitin ligases^16,17^. The E3 ubiquitin ligase CHIP is believed to participate in the protein triage decision by preferentially ubiquitinating chaperone-bound substrates^12,13,17^. To further understand the regulation process, the influence of GRP75 on CHIP recruitment to the Hsc70/Hsp90/proto-Dbl complex was investigated. GST-tagged proto- or onco-Dbl plasmids were co-transfected with myc-tagged CHIP plasmids in GRP75 knockdown Cos-7 cells. As expected, GST-proto-Dbl was directly ubiquitinated by the exogenous expression of CHIP (Supplementary Fig. 5), which suggests that proto-Dbl is a direct substrate of the ubiquitin E3 ligase CHIP. Again, the pull-down results showed that GRP75 knockdown weakens the ubiquitin modification of proto-Dbl (Supplementary Fig. 5). Notably, the co-pull-down results showed that GRP75 knockdown markedly increased the binding of Hsc70 and Hsp90 but significantly decreased CHIP recruitment to proto-Dbl in the pro-degradation complex (Fig. 6a). This GRP75 expression intervention-induced Hsc70/Hsp90 binding increased at the expense of decreased CHIP binding markedly attenuated the degradation of proto-Dbl. Similar results were observed when exogenous myc-CHIP (or endogenous CHIP) was co-immunoprecipitated by GST-proto-Dbl Ab (or Dbl Ab) in co-transfected Cos-7 cells (or in SKOV-3 cells) (Fig. 6b, c). Taken together, these results suggest that GRP75 promotes proto-Dbl degradation by regulating multi-components (reinforcing CHIP recruitment and competing with Hsc70/Hsp90) in the ubiquitin–proteasome machinery (Fig. 8).

### GRP75 inhibition downregulates onco-Dbl-induced RhoGTPases activation

**Fig. 7:**
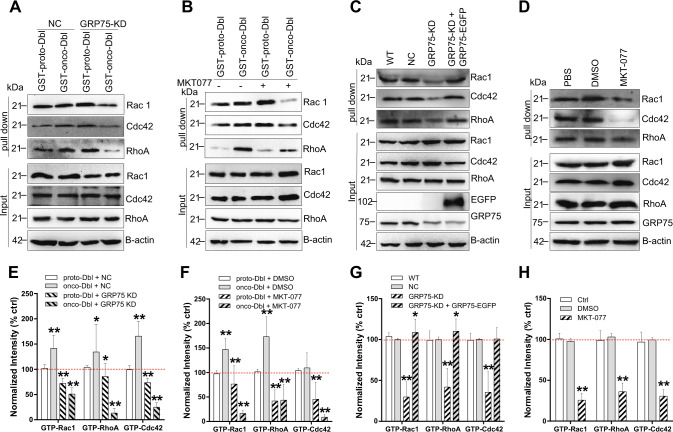
GRP75 knockdown or inhibition decreases onco-Dbl-induced Rho GTPases activation. **a** GRP75 KD (G3) Cos-7 cells were transfected with either GST-proto-Dbl or GST-onco-Dbl plasmids and cultured for 32 h. The cells were then serum-starved for 16 h, lysed, and incubated with GST-fused CRIB (Cdc42 and Rac1 activation) or GST-fused TRBD (RhoA activation) immobilized sepharose beads. The activated Rho GTPases in pull-down precipitates were immunoblotted with corresponding Abs. **b** COS-7 cells were transfected with GST-Dbl plasmids, serum-starved as described above, and then treated with MKT077 (40 µM, 6 h) before harvest. The activation level changes of Rho GTPases were checked as described above. **c** GRP75 KD (G3) SKOV-3 cells were transfected with GRP75-EGFP plasmids. SKOV-3 (wt, ctrl), NC SKOV-3, GRP75 KD (G3), and GRP75-EGFP rescued GRP75 KD (G3) cells were serum-starved, lysed, and pull-down for activated Rho GTPase checking as described above. **d** SKOV-3 (wt, ctrl) cells were treated either with DMSO or with MKT077 (40 µM, 6 h), serum-starved, lysed, and pulled-down for activated Rho GTPase checking as described above. Representative blot results from three independent experiments are shown in **a**, **b**, **c**, **d**. Protein brands were quantified by Image J software. The activation variation of Rho GTPase proteins were quantified and shown in **e**, **f**, **g**, **h**, respectively. Statistically significant differences compared with corresponding ctrls (**e**), proto-Dbl transfected NC Cos-7 cells; **f**, DMSO treated Cos-7 cells; **g**, **h**, untransfected SKOV-3 cells (WT or Ctrl)) are shown: **P < 0.01, *P < 0.05

As distinct endocytosis pathways are dependent on different small GTPases^5,33^, which are activated by upstream Dbl family GEFs^8,9,27^, we then speculated that knockdown or inhibition of GRP75 attenuated proto-Dbl degradation and reduced the onco-Dbl level, which could cause the Rho GTPase activation to decrease. Actually, in GST-onco-Dbl ectopically expressed Cos-7 cells, both GRP75 knockdown and its inhibition by MKT077 significantly decreased the activation of RhoA, Cdc42, and Rac1 (Fig. 7a, b, e, f). In contrast, knockdown or inhibition of GRP75 in GST-proto-Dbl expressed Cos-7 cells caused a little activation decrease of the three Rho GTPases (Fig. 7a, b, e, f). When checking their activation changes in SKOV-3 cells, GRP75 knockdown or its inhibition induced Rac1, RhoA, and Cdc42 activations to be similarly downregulated (Fig. 7c, d, g, h). Notably, in SKOV-3 cells and in onco-Dbl-transfected Cos-7 cells, GRP75 knockdown (or its inhibition) more significantly decreased the activations of Rac1 and RhoA than that of Cdc42 (Fig. 7). Hence, these results together with the aforementioned observations collectively suggest that GRP75 controls RhoGTPases activation by regulating ubiquitin–proteasome-mediated proto-Dbl degradation. The striking difference in the activation level of Rac1, RhoA, and Cdc42 largely affects the balance of intracellular endocytosis networks.

## Discussion

Multiple evidence supports oncogenic alteration contributing to cellular endocytosis derailment. Early studies showed non-receptor kinase v-Src overexpression or c-Src^Y527F^ activated-mutation promotes the fluid-phase uptake in transformed fibroblasts and HeLa cells^34–36^. More studies showed oncogenic RAS-dependent endocytosis changed. H-Ras activation, either through EGF stimulation or due to H-Ras^G12V^ mutation, enhanced macropinocytosis activity in HeLa, COS-7, MCF7, and CHO cells^37–39^. Notably, macropinocytosis activated by H-Ras is dependent on the amount of oncogenic protein, but also dependent on its intracellular longevity/persistence. Furthermore, K-Ras^G13V^ activated-mutation intensively increased CaME of polyamine in colon cancer cells. K-Ras^G12D^ activated-mutation markedly increased the macropinocytosis uptake of extracellular protein, exosomes, iExosomes, and recombinant β-defensin in pancreatic cancer cells^40–44^. We found for the first time that oncogenic Dbl expression significantly promotes macropinocytosis activity (Figs. 1 and 2, Supplementary Figs. 3, 4), and the promotion effect induced by onco-Dbl is more potent than that by proto-Dbl (Fig. 2, Supplementary Figs. 3h, 4h). Our result is in line with a previous report that pinocytosis activity induced by proto-H-Ras is less than that by onco-Ras [38], reinforcing the high potential of the oncogenic protein on the endocytosis-derailed phenotype.

Actually, Dbl is not the only Dbl family member exerting an endocytosis regulatory function. Previous studies showed other Dbl family proteins, such as Tiam-1, ITSNs, Vav2, also function in endocytosis regulation^45–47^. The endocytosis regulatory mechanism of these proteins may be that most of them are oncogenic GEFs specific for certain Rho GTPase activation, which mediate various signaling including cytoskeleton reorganization-based membrane trafficking^27^. As a Rac1-specific GEF, Vav2 expression delayed the CME of EGFR through interacting with endosome-associated proteins Gapvd1 and Tom1L1^48^. knockdown of Tiam-1, a Rac2-specific GEF, resulted in inefficient CME of EphA8-ephrinA5 complexes^49^. Whereas, silencing of ITSN1/2, specific GEFs for Cdc42, slowed both CME and CaME^50^. We found in this study that Dbl expression enhanced macropinocytosis but simultaneously inhibited CME and lipid-raft endocytosis (Figs. 2 and 3, Supplementary Figs. 3, 4). This induced phenotype differs from the aforementioned Dbl family proteins but is similar to the Src-induced disparate change in endocytosis, by which macropinocytosis is elevated whereas CME is poorly affected^35,36^. Why oncogenic Dbl expression only elevates the macropinocytosis pathway but inhibites other endocytosis pathways is not clear. One reason could be that, like partial Dbl family members^51–53^, mutation-truncated Dbl feedback activates the upstream Src tyrosine kinase, which can phosphorylate the clathrin heavy chain and therefore leads to CME impairment^34^. Another reason may be that Rac1 is mostly activated by onco-Dbl (compared with RhoA and Cdc42 activations. Figure 7), which generally induced intensive macropinocytosis in cancer cells^5,6,44,51^.

**Fig. 8:**
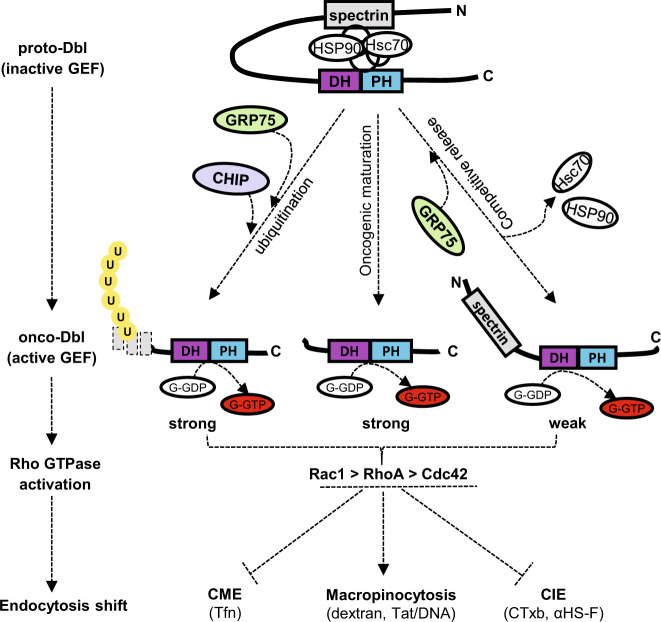
Schematic model of GRP75-Dbl signaling axis regulates endocytosis derailment. Current results of GRP75 involved in the regulation of proto-Dbl activities are summarized together with previous published data^10,18^. See Discussion for details

It may be argued that Dbl GEF theoretically has a different catalytic efficiency towards Rho GTPases (RhoA > Cdc42 > Rac1)^27^, which may conflict with the major role of Rac1 in onco-Dbl-induced macropinocytosis enhancement. As the macropinocytosis pathway is actin polymerization-dependent and dynamically triggered by reciprocal activation of different Rho GTPases^54^. No matter which one is the primary GTPase activated by oncogenic Dbl, macropinocytosis is the most likely resulting endocytosis route. Of course, some researchers may insist that Dbl GEF exhibits higher selectivity to RhoA and Cdc42 than to Rac1^7,27^ and only the former two Rho GTPases activation-dependent endocytosis pathways could be motivated by onco-Dbl action^6,33^. As we previously found that the ectopically expressed GRP75 reduces CME but upregulates CIE^25^ and others ever documented that RhoA and Rac1 activations inhibit CME^54^, the dominant macropinocytosis co-regulated by Dbl and GRP75 is also possible (Fig. 8).

CHIP is a master player with dual functions both as a co-chaperone and an E3 ubiquitin ligase in the cellular triage decision. The N-terminal TPR domain in CHIP mediates its interaction with the C-terminal EEVD domain in both Hsp70/Hsc70 and Hsp90. CHIP modulates the chaperone function by blocking the ATPase cycle and inhibiting the folding activity of the chaperone protein. The C-terminal of CHIP contains a U-box domain with E3 ubiquitin ligase activity. CHIP acts as a scaffold that positions the chaperone’s substrate near the Ubc4/5 family stress-associated E2 ubiquitin-conjugating enzyme. The proteostasis and longevity of the substrate are thus controlled by the level of the CHIP E3 ubiquitin ligase. Actually, the CHIP protein level is strictly held by certain chaperones in a given cell context. Early studies showed that proteasome-mediated degradations of the Parkinson’s disease (PD) causative ono-protein DJ-1 and tumor suppressor P53 are facilitated by the CHIP-GRP75 complex^55–57^. Overexpression of UBXN2A, an inhibitory protein against GRP75, disrupts the formation of the GRP75-CHIP complex and consequently suppresses the CHIP-mediated destabilization of p53^55^. We found in this study that GRP75 knockdown or inhibition suppresses the ubiquitination and degradation of both exogenous GST-proto-Dbl in Cos-7 cells and endogenous proto-Dbl in ovarian cancer cells (Figs. 4, 5c, d and 6). These suppression effects were largely due to GRP75 knockdown/inhibition which reduces the amount of CHIP recruited to the proto-Dbl folding complex (Fig. 6a, b), suggesting their cooperative relation in the pro-degradation process. On the other hand, we also detected the increased association of Hsc70 and Hsp90 to this complex after GRP75 attenuation (Fig. 6a), suggesting the competing relations between GRP75 and Hsc70/Hsp90 in the pro-folding process. Together, these results suggest that the CHIP protein level is controlled by expression of GRP75, and GRP75 is a competitor to Hsc70/Hsp90 in the CHIP-mediated polyubiquitination of proto-Dbl (Fig. 8).

Despite previous studies suggesting that CHIP E3 ubiquitin ligase could associate with GRP75 and mediate proteasome degradation of certain substrates^55–57^, there is no evidence showing the direct binding of CHIP to GRP75. We purposefully checked whether GRP75 directly interacts with CHIP, and no direct binding of GRP75 to both endogenous CHIP and exogenous myc-CHIP were detected in co-IP assays (Supplementary Fig. 6). These results are not in accordance with the previous observation that GRP75 could bind with three other E3 ubiquitin ligases. One is mitochondria-localized parkin, shown to be selectively recruited and interacting with GRP75 in impaired mitochondria, and therefore mediating polyubiquitination of VDAC (the voltage-dependent anion channel)^58–60^. The other two are AIP4/Itch and AIP5/WWP1, confirmed to bind with GRP75 and playing roles in the protein turnover of the Troyer syndrome causative protein Spartin^61^. Notably, we found that GRP75 knockdown downregulated the protein level of endogenous CHIP (Fig. 6c), which implies that beyond the GRP75 cooperation-mediated CHIP level control in the chaperone-folding complex, there might be a GRP75-mediated signaling pathway controlling the cytoplasmic CHIP level. This signaling mechanism needs to be investigated in the future.

In summary, our study presents a role of GRP75 in the Dbl-driven endocytosis-derailed phenotype. This finding not only uncovers a novel signaling axis that regulates the cellular endocytosis network, but also provides an alternative using a chaperone inhibitor to shut down the oncoprotein-activated macropinocytosis route.

## Materials and methods

### Cell culture, antibodies, plasmids, and reagents

SKOV-3, Cos-7, and 293T cell lines obtained from Type Culture Collection of Chinese Academy of Sciences (China), were maintained in DMEM containing 10% (v/v) fetal bovine serum. Mouse anti-GRP75 Ab (sc-133137), anti-Hsc70 Ab (sc-7298), anti-Hsp90 Ab (sc-13119), anti-Dbl Ab (sc-89), anti-GST Ab (sc-138), anti-CHIP Ab (sc-133066), anti-Cdc42 Ab (sc-8401), anti-RhoA Ab (sc-418), and anti-Rac1 Ab (sc-514583) were obtained from Santa Cruz. Rabbit anti-GFP Ab (D110008), anti-myc Ab (AB10006), and mouse anti-β-actin Ab (D190606) were obtained from Sangon Biotech. Tfn-AF647 (T-23366), CTxB-AF647 (C-34778), goat anti-mouse Ab-AF488 (A-11001), goat anti-mouse Ab-AF647 (A32728), goat anti-rabbit Ab-AF488 (A-11070), Lipofectamine 3000, Dynabeads protein G (10003D), and GSH Glutathione sepharose 4B (17-0756-10) were obtained from Life technologies. The lentiviral CRISPR/Cas9 system was obtained from Addgene. Anti-HS scFv Ab (AO4B08) was kindly supplied by Prof. Toin H. van Kuppevelt (Nijmegen Centre for Molecular Life Sciences). proto-Dbl (residues 1–925), onco-Dbl (residues 498–925), and N-Dbl (residues 1–498) were GST-N-terminal cloned into pEBG plasmids (GST-P) and kindly supplied by Prof. Danny Manor (Case Western Reserve University)^10^. CHIP-myc, CHIP ΔTPR-myc, CHIP ΔU-box-myc, and ubiquitin-HA plasmids (pcDNA3) were kindly supplied by Prof. Shigeo Murata (The University of Tokyo). The GRP75-EGFP plasmid was previous constructed^25^. Mouse anti-VSV Ab P5D4 (V5507), MKT077 (M5449), RO3306 (SML0569), Hochest33342, polybrene (H9268), and puromycin (P7255) were obtained from Sigma. MG132 (M126521) was obtained from Aladdin Biotech.

### Magnetic vesicle purification

The process was similar to that previously described^24,32^. Briefly, αHS scFv (1:40), mouse anti-VSV (1:500), and magnetic MagCellect goat anti-mouse (1:20) antibodies were allowed to form complexes (scFv-αHSM) in DMEM at 20 °C for 30 min. scFv-αHSM was then added to cells at 37 °C for 1 h, followed by washing with PBS, trypsin detachment, suspension in DMEM 10% FBS, and washing with PBS. The complete protease inhibitor (Roche) was included in all subsequent steps. Cells were then mechanically disrupted by passage through a 27-G needle during 25 strokes. The remaining intact cells, cell debris, and nuclei were removed by centrifugation at 500 × *g* for 5 min. The resulting supernatant was pooled with the supernatant obtained after a second centrifugation at 300 × *g* for 3 min to yield a post-nuclear supernatant (PNS), which was separated into a magnetic and nonmagnetic fraction using a magnetic separator (PickPen; Bio-Nobile) (Supplementary Fig. 1). Protein samples in isolated vesicles were quantified for the subsequent steps.

### Mass spectrometry identification

The process was as previously described^24,62^. Briefly, vesicle-derived protein samples were separated by 1D SDS-PAGE in duplicate. One gel was silver stained, the other gel was Coomassie blue stained, and the specific protein bands were excised. Bands of interest were washed using 40% acetonitrile, speedVac dried, and in-gel digested with trypsin at 37 °C overnight. After 1 h extraction by 2% trifluoroacetic acid, peptides were purified using C18 reversed-phase tips, spotted directly onto a stainless steel MALDI target, and left to dry. A matrix solution containing 5 mg/ml a-cyano-4-hydroxycinnamic acid, 50% acetonitrile, 0.1% trifluoroacetic acid, and 50 mM citric acid was added and allowed to dry, and MS/MS spectra were acquired using a 4700 Proteomics Analyzer (Applied Biosystems, Framingham, CA) in positive reflector mode. Protein identification was performed using the GPS Explorer software, with an in-house Mascot search engine searching the NCBI non-redundant database.

### Construction of stable knockdown cell lines and plasmid overexpression

For long-term gene silencing, the lentiviral CRISPR/Cas9 system was used to produce GRP75 and Dbl stable knockdown cell lines. The GRP75-targeting sgRNA constructs were generated as previously described^26^. Three independent Dbl-targeting sgRNA constructs were generated by ligation of annealed oligonucleotides, which targeted different regions of human Dbl mRNA (sgRNA#1: GCAACGAACGTTCACAGACAT, sgRNA#2: GGCAGAAGCAAATCCCCGGAG, and sgRNA#3: GGCAGAACTGGCTGATGTAAC), into the pLenti-CRISPR v2 plasmid, respectively. For lentivirus production, 293T cells were co-transfected by recombinant pLenti-CRISPR v2 plasmids together with psPAX2 and pCMV-VSV-G packaging plasmids, as described in the manual. Viral supernatants were collected after 72 h and mixed with polybrene (4 μg/ml) before use. The optimal puromycin concentration (SKOV-3 5 μg/ml and COS-7 0.5 μg/ml) was used to select stably transduced cell lines.

For gene overexpression in Cos-7 or SKOV-3 cells, Dbl or CHIP plasmids alone, or combined with ubiquitin or CHIP plasmids were transiently transfected by Lipofectamine®3000 according to the instructions. Specific knockdown or overexpression of indicated proteins was confirmed by Western blot using corresponding Abs.

### Uptake measurement by confocal imaging and flow cytometry analysis

The process was as previously described^25,26^. Briefly, the incubation conditions (final conc. and treated time) for endocytosis markers (Tfn-AF647, 25 µg/ml, 15 min; Dextran-Rhodamine, 4 mg/ml, 1 h; CTxB-AF647, 10 µg/ml, 30 min), scFv-αHS_F_ (AF647-labeled scFv-αHS complexes, 1:20) and Tat/pGL3-YOYO-1 complexes (10ug pDNA/ml, 1 h) were first optimized. Transfected or stable knockdown cells were incubated with fluorescent-labeled ligands at 37 °C, twice rinsed with PBS/0.5 M NaCl, and immediately image-acquired using an Olympus FV 1000 confocal microscope. The binding and uptake measurements were performed as previously described^25,26^. Trypan Blue was added to quench cell surface-associated fluorescence before cells were harvested for immediately FACS analysis. Software-based quantification analysis of intracellular fluorescent particles was performed as previously described^25,26^. To compare the uptake in cultures with drug inhibition, medium conditions were achieved in cells via incubation with the GRP75 inhibitor (MKT077, 40 µM final concentration of DMSO was <0.4%).

### GST-pull-down, Co-IP, and Western blotting

Forty-eight hours post-transfection, cells were lysed with a lysis buffer (20 mM HEPES, pH 7.4, 150 mM NaCl, 1% NP-40, 20 mM sodium fluoride, 1 mM EDTA, 1 mM activated sodium vanadate). The protein concentration of the lysis supernatant was measured using the BCA assay. Samples were incubated with GSH Glutathione sepharose 4B overnight at 4 °C. The beads were washed 5–6 times with lysis buffer. Bound proteins were boiled with 2× loading buffer, resolved on SDS-PAGE, and blotted with corresponding Abs. Immunoprecipitations were similarly performed by using the indicated antibodies. Immunoprecipitated proteins were immobilized on protein A/G-agarose, washed, and subjected to Western blotting. The blotting Abs dilution was as follows: anti-Dbl Ab (1:200), anti-GRP75 Ab (1:500), anti-GST Ab (1:500), anti-myc Ab (1: 1000), anti-CHIP Ab (1:500), anti-Cdc42 Ab (1:250), anti-RhoA Ab (1:250), anti-Rac1 Ab (1:200), and anti-B-actin Ab (1:2000).

### Rho GTPase activation assays

GTPase activation assays were created following the GST-RBD (Rho-binding domains) pull-down method as previously described^25^. Briefly, serum-starved cells were lysed and the pre-cleared cell lysate was incubated with GST-PAK-CRIB beads (for Cdc42/Rac activation) or GST-Rhotekin-RBD beads (for RhoA activation). After subjecting the collected pellets to SDS-PAGE, the GTP-bound Cdc42, RhoA, and Rac1 in samples were determined by Western blotting.

### Statistical analyses

Confocal microscopy, flow cytometry, and Western blotting data were derived from at least three independent experiments. All of the data obtained from the experiments were analyzed and are presented as the mean ± SD. For two-sample comparisons against the controls, unpaired Student’s *t*-tests were used unless otherwise noted. One-way analysis of variance with a Dunnett’s multiple comparisons was used to evaluate the statistical significance of at least three groups of samples. Graphs were created using GraphPad Prism 5 software.

## Electronic supplementary material

Supplyment files
