# Supplementary material for: GRP75 modulates oncogenic Dbl-driven endocytosis derailed via the CHIP-mediated ubiquitin degradation pathway
Source: Cell Death Dis. 2018 Sep 24;9(10):971. doi: 10.1038/s41419-018-1039-2 (PMC6155137; doi:10.1038/s41419-018-1039-2)
Supplement: Supplementary file 1 — Supplyment files [file 41419_2018_1039_MOESM1_ESM.docx]

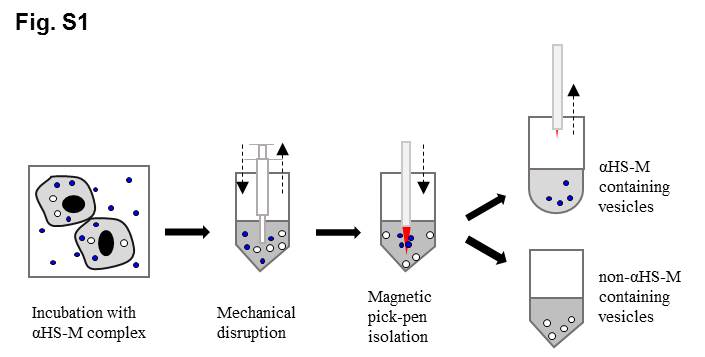


**Supplemental Fig. 1 Magnetic isolation of aHS-M nanoparticles containing endocytosis vesicles.** Cells were incubated with scFv-αHS-M nanoparticles (visualized in blue). After uptake for a given period of time, cell surface associated aHS-M nanoparticles were removed by trypsinization and extensive washing. Cells were then mechanically disrupted by a syringe (shearing with the 27G needle), nuclei and cell debris were removed to yield a PNS fraction consisting both aHS-M nanoparticles containing vesicles and other cellular components. After purification by using a magnetic pick-pen, the PNS fraction was separated into a magnetic fraction with aHS-M nanoparticles containing vesicles and a non-magnetic fraction with the remaining cellular components.


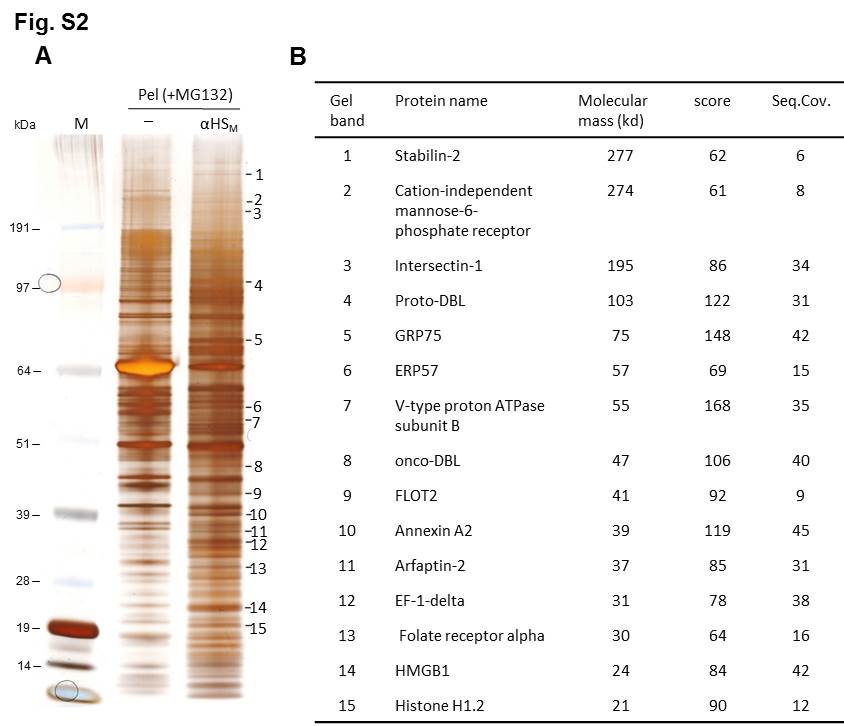


**Supplemental Fig. 2 Mass spectrometry identification of vesicle-enriched proteins. a** The Pel nonmagnetic (-) and magnetic (aHS-M) fractions from the experiment described in ‘Materials and methods’ silver stained and analyzed by 1D gel electrophoresis showed a distinct pattern of protein bands in the aHS-M fraction. **b** Major discrepant bands from the corresponding gel (Coomassie Blue staining) were excised and analyzed by mass spectrometry. The identities of vesicular proteins are shown in the Table . Seq. cov., sequence coverage. Gel band numbers correspond to the numbers in (A)**.**

**
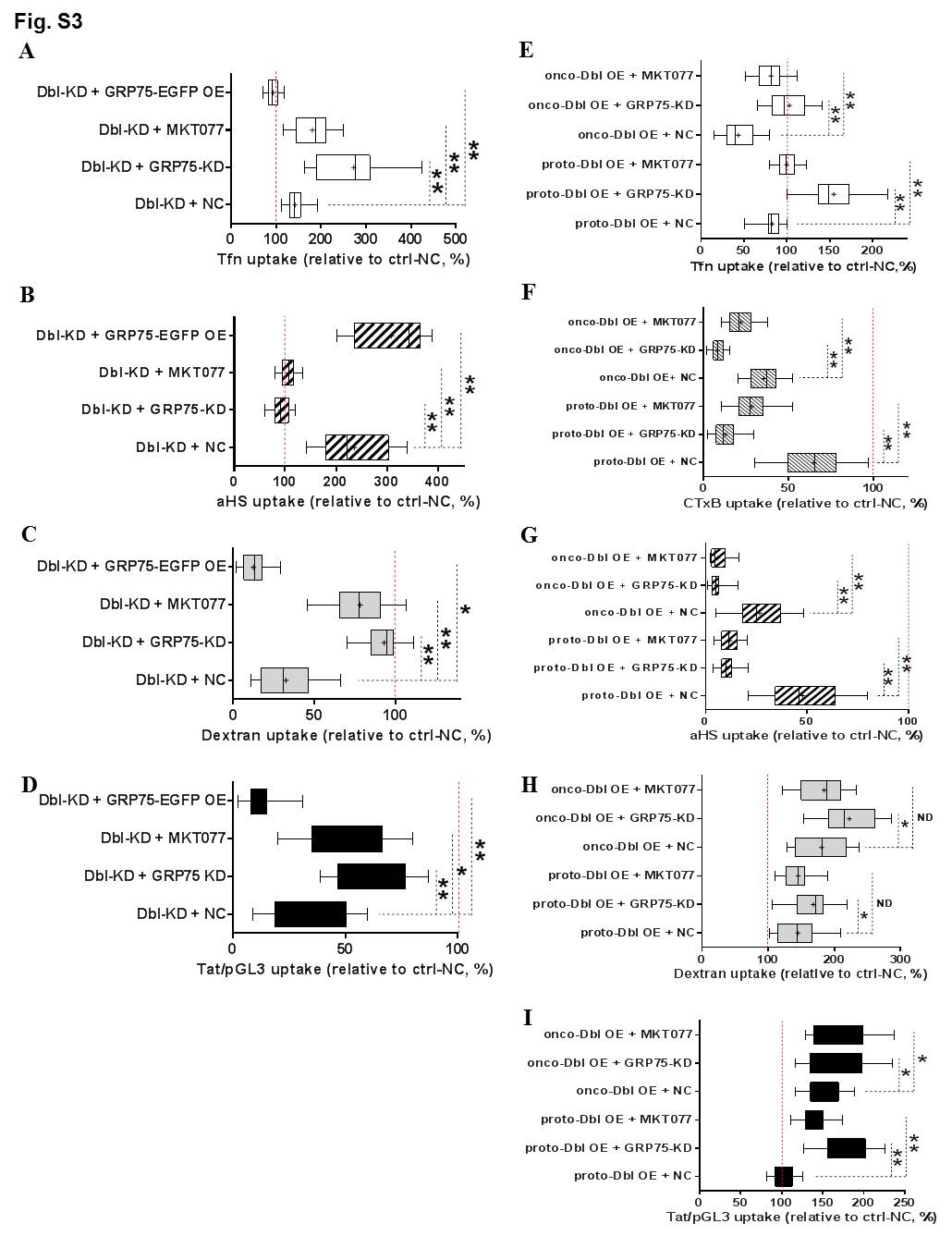
**

**Supplemental Fig. 3 Confocal imaging analyzed the uptake variability of fluorescent-labeled drugs.** **a**, **b**, **c**, **d** Box-and-whiskers plots depict the uptake of fluorescent-labeled drugs in SKOV-3 cell populations. Uptake levels in Dbl knock-down, Dbl & GRP75 double knock-down, Dbl knock-down followed by MKT077 treatment, or Dbl knock-down followed by GRP75-EGFP overexpression (labeled with rabbit anti-GFP Ab followed by anti-rabbit Ab-AF488 staining, or by anti-rabbit Ab-AF647 staining (for the Tat/pGL3-YOYO-1 group)) SKOV-3 cells were quantified as described in ‘Materials and methods’. The uptake in NC SKOV-3 cells (ctrl-NC) was set as 100% for comparison. ≥60 cells were counted for each transfection, n=3. Significant differences compared with Dbl knock-down NC SKOV-3 cells (Dbl-KD+NC) are shown: ** P <0.01, * P <0.05. **e**, **f**, **g**, **h**, **i** Box-and-whiskers plots depict the uptake variability of fluorescent-labeled drugs in Cos-7 cell populations. Uptake levels in GST-fusion expressed Cos-7 cells (labeled with mouse anti-GST Ab followed by anti-mouse Ab-AF488 staining, or by anti-mouse Ab-AF567 staining (for the Tat/pGL3-YOYO-1 group)) were quantified as described in ‘Materials and methods’. The uptake of pEBG-GST transfected NC Cos-7 cells (ctrl-NC) was set as 100% for comparison. ≥60 cells were counted for each transfection, n=3. Significant differences compared with proto-Dbl transfected NC Cos-7 cells (proto-Dbl OE+NC) are shown: ** P <0.01, * P <0.05.


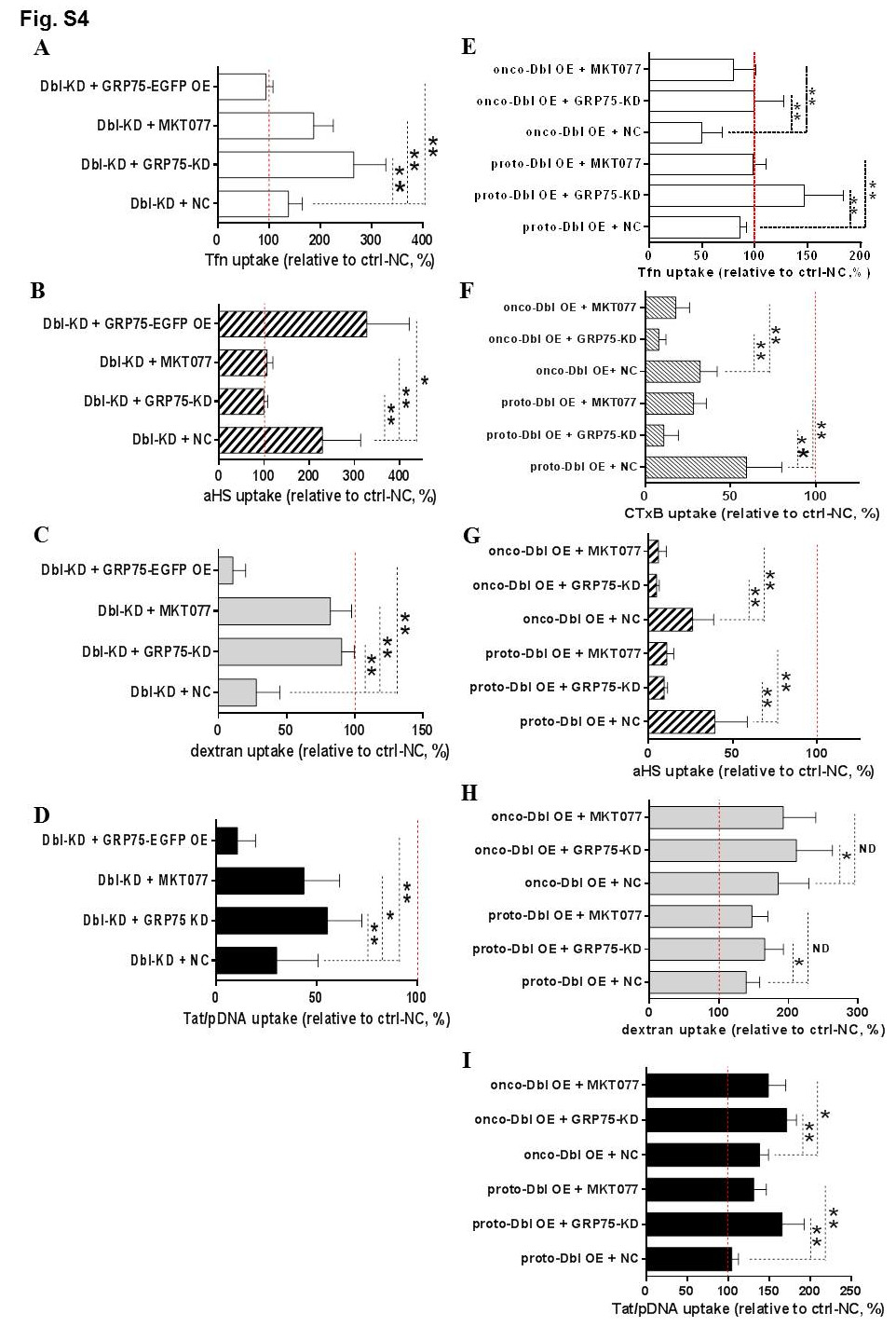


**Supplemental Fig. 4 Flow cytometry analyzed the uptake variability of fluorescent-labeled drugs.** **a**, **b**, **c**, **d** The uptake levels of fluorescent-labeled drugs (Tfn-AF647, CTxB-AF647, aHS-AF647, Dextran-Rhodamine, and Tat/pGL3-YOYO-1) in Dbl-KD (D3) SKOV-3 cells were analyzed and quantified by flow cytometry. ctrl-NC, DMSO-treated NC SKOV-3 cells. 10,000 cells were counted per sample with triplicate samples per transfection for each experiment, n=3. Statistically significant differences compared with Dbl-KD+NC (NC lentivirus-treated Dbl-KD (D3) SKOV-3 cells) are respectively shown: ** P <0.01, * P <0.05. **e**, **f, g, h, i** The uptake levels of indicated fluorescent-labeled drugs in transfected Cos-7 cell populations were similarly analyzed by flow cytometry as described above. GRP75-KD, GRP75 knock-down (G3) Cos-7 cells. ctrl-NC, pEBG-GST plasmids transfected NC Cos-7 cells. Statistically significant differences compared either with proto-Dbl OE+NC (proto-Dbl overexpressed NC Cos-7 cells) or with onco-Dbl OE+NC (onco-Dbl overexpressed NC Cos-7 cells) are shown: ** P <0.01, * P <0.05.


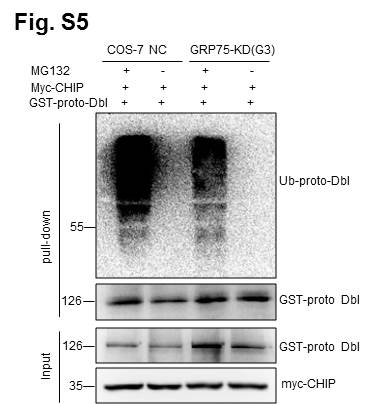


**Supplemental Fig. 5 proto-Dbl is a direct substrate of the ubiquitin E3 ligase CHIP.** Cos-7 NC and GRP75 KD (G3) stable cell lines were co-transfected with GST-proto-Dbl and myc-CHIP plasmids for 40h culture, and then treated with/without MG132 (25μM) for 6h. Exogenous Dbl was pulled-down from cell lysate by glutathione sepharose 4B. Ectopically expressed GST-proto-Dbl and myc-CHIP were determined by Western blot using mouse anti-GST Ab and rabbit anti-Myc Ab, respectively. ubiquitinated GST-proto-Dbl in the pull-down pellet was blotted by mouse anti-ubiquitin (Ub) Ab.

**
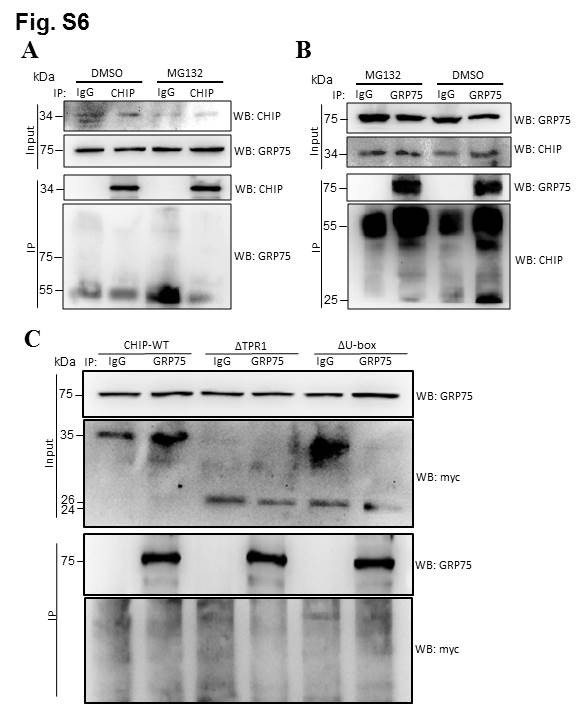
**

**Supplemental Fig. 6 GRP75 does not bind with CHIP**. **a**, **b** Endogenous GRP75 and CHIP from SKOV-3 (with/without MG132 treatment) cell lysates were co-immunoprecipitated in a bi-directional manner. Proteins precipitates were subjected to Western blot analysis using the indicated Abs. The isotype-matched IgG was used as the control; **c** Cos-7 cells were transfected with different CHIP constructs (myc-CHIP WT, myc-CHIP ΔU-box, myc-CHIP ΔTPR,). The ectopically expressed CHIP protein or its truncated mutations was co-immunoprecipitated by mouse anti-GRP75 Ab. Proteins precipitates were subjected to Western blot analysis using the indicated Abs. The isotype-matched IgG was used as the control.
